# Differences in vulnerability of neurons and astrocytes to heme oxygenase-1 modulation: Implications for mitochondrial ferritin

**DOI:** 10.1038/srep24200

**Published:** 2016-04-21

**Authors:** Xiaojun Yu, Ning Song, Xinli Guo, Hong Jiang, Haoyun Zhang, Junxia Xie

**Affiliations:** 1 Collaborative Innovation Center for Brain Science, Department of Physiology, Shandong Provincial Collaborative Innovation Center for Neurodegenerative Disorders, Key Laboratory of Pathogenesis and Prevention of Neurological Disorders and State Key Disciplines: Physiology, Medical College of Qingdao University, Qingdao, 266071, China

## Abstract

Induction of the antioxidant enzyme heme oxygenase-1 (HO-1) was observed in both astrocytes and neurons in the substantia nigra of patients with Parkinson’s disease (PD). In the current study, we investigated whether HO-1 behaves differently between neurons and astrocytes under the condition of neurotoxicity related to PD. The results showed a time-dependent HO-1 upregulation in primary cultured ventral mesencephalon neurons and astrocytes treated with the mitochondria complex I inhibitor 1-methyl-4-phenylpyridinium (MPP^+^) or recombinant α-synuclein. However, HO-1 upregulation appeared much later in neurons than in astrocytes. The HO-1 inhibitor zinc protoporphyrin (ZnPP) aggravated MPP^+^- or α-synuclein-induced oxidative damage in both astrocytes and neurons, indicating that this HO-1 response was cytoprotective. For neurons, the HO-1 activator cobalt protoporphyrin IX (CoPPIX) exerted protective effects against MPP^+^ or α-synuclein during moderate HO-1 upregulation, but it aggravated damage at the peak of the HO-1 response. For astrocytes, CoPPIXalways showed protective effects. Higher basal and CoPPIX-induced mitochondrial ferritin (MtFt) levels were detected in astrocytes. Lentivirus-mediated MtFt overexpression rescued the neuronal damage induced by CoPPIX, indicating that large MtFt buffering capacity contributes to pronounced HO-1 tolerance in astrocytes. Such findings suggest that astrocyte-targeted HO-1 interventions and MtFt modulations have potential as novel pharmacological strategies in PD.

Parkinson’s disease (PD) is a common neurodegenerative disorder characterized by progressive degeneration and loss of dopaminergic neurons in the substantia nigra pars compacta (SNpc). PD is associated with several risk factors that include advanced age, genetic susceptibility and environmental factors, and these factors can act synergistically or additively to promote degeneration of dopaminergic neurons^1^,^2^,^3^,^4^,^5^. Based on these risk factors, oxidative stress is one of the most common mechanisms in the pathogenesis of PD^6^,^7^. Indeed, the most commonly used therapy for PD, levodopa, is a pro-oxidant and causes oxidative stress^8^.

Dopaminergic neurons are particularly vulnerable to oxidative stress. This vulnerability might be due to but not limited to the conversion of excess dopamine into dopamine quinone or hydrogen peroxide as well as the presence of enriched iron in the substantia nigra^9^,^10^,^11^,^12^. Reactive astrocytes were associated with neuronal loss in the SNpc; however, glial cells display significantly greater resistance to oxidative stress-induced neurotoxicity^13^,^14^,^15^. A large cell-buffering system, such as the glutathione (GSH) synthesis system, might aid in counteracting damage in astrocytes caused by reactive oxygen species (ROS)^15^,^16^,^17^. Therefore, astrocytes are a potential candidate for anti-oxidant intervention in PD.

Two genetically distinct isoforms of heme oxygenase (HO) have been described: an inducible form, HO-1, and a constitutively expressed form, HO-2^18^. Whereas HO-2 regulates normal physiological cell function, HO- 1, which is also known as heat shock protein 32, is an inducible enzyme that is considered a measurable indicator of oxidative stress. Intracellular HO-1 induction provides a host defense mechanism against oxidative injury caused by substances including neurotoxins, glutamate, and hydrogen peroxide^19^,^20^,^21^,^22^. The HO family catalyzes the rate-limiting step of oxidative degradation of cellular heme into carbon monoxide (CO), bilirubin and free iron^23^. CO and biliverdin serve as powerful reductive byproducts. However, free iron produced during this catalytic process aggravates cell damage via the Fenton reaction^24^,^25^,^26^. This two-sided effect implicates HO-1 as a controversial molecule that acts as a double-edged sword in cell activity and survival^23^.

Intense HO-1 immunoreactivity was observed in both Lewy bodies and glial fibrillary acidic protein (GFAP)-positive astrocytes in the SNpc of PD patients. In other brain regions, HO-1 staining remained unchanged or was faint and localized to small, scattered groups of neurons and glia^27^. However, the mechanisms underlying HO-1 actions in neurons and astrocytes remain unknown. In the current study, we used the mitochondria complex I inhibitor 1-methyl-4-phenylpyridinium (MPP^+^) and investigated whether HO-1 behaves distinctively between neurons and astrocytes under the condition of neurotoxicity related to PD in primary cultured ventral mesencephalon (VM) neurons and astrocytes. Recombinant α-synuclein was also used in this study because it can propagate to neighboring cells and contribute to neuronal degeneration in PD^28^,^29^,^30^. We also aimed to elucidate the mechanisms underlying the differences in vulnerability to HO-1 modulation between neurons and astrocytes.

## Results

### Upregulation of HO-1 induced by MPP^+^ or recombinant α-synuclein occurs much earlier in VM astrocytes than in neurons

**Figure 1:**
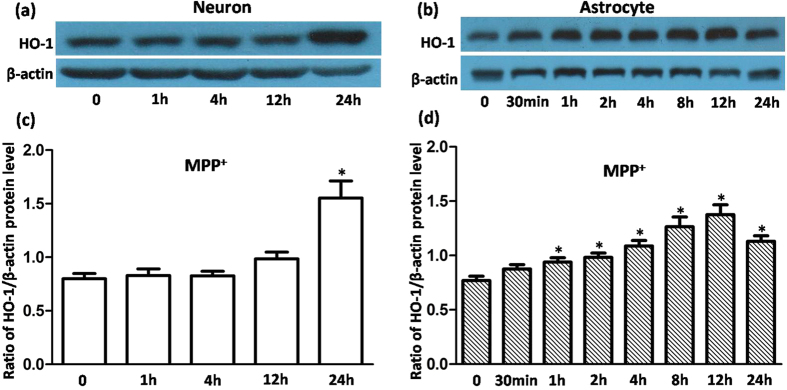
HO-1 protein levels were upregulated in VM neurons and astrocytes treated with MPP^+^. (**a**,**b**) Western blots were used to detect HO-1 protein levels in cells with 100 μmol/L MPP^+^ treatment. HO-1 expression in neurons tended to increase at 12 h, but significant changes were observed only at 24 h. For astrocytes, HO-1 upregulation was detected at 1 h, 2 h, 4 h, 8 h, 12 h, and 24 h. β-Actin was used as a loading control. (**c**,**d**) Statistical analysis. Data are presented as the ratio of HO-1 to β-actin. Each bar represents the mean ± S.E.M. of more than 6 independent experiments. ^*^P < 0.05, compared with control.

**Figure 2:**
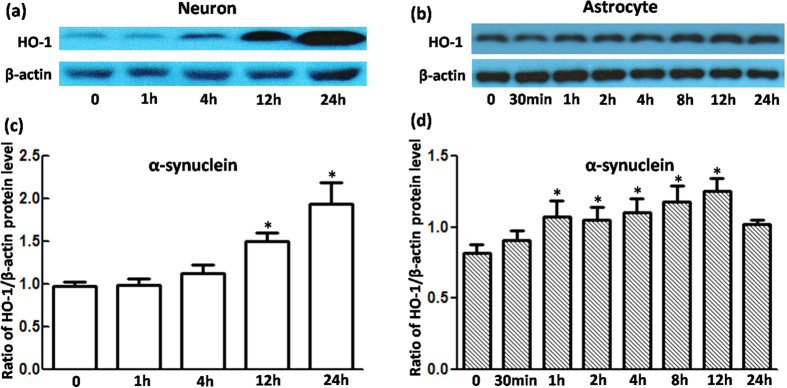
HO-1 protein levels were upregulated in VM neurons and astrocytes treated with α-synuclein. (**a**,**b**) Western blots were used to detect HO-1 protein levels in cells with 5 μg/mL α-synuclein treatment. HO-1 expression in neurons increased at 12 h and 24 h. For astrocytes, HO-1 upregulation was detected at 1 h, 2 h, 4 h, 8 h, 12 h, and 24 h. β-Actin was used as a loading control. (**c**,**d**) Statistical analysis. The data are presented as the ratio of HO-1 to β-actin. Each bar represents the mean ± S.E.M. of more than 6 independent experiments. ^*^P < 0.05, compared with control.

To elucidate whether HO-1 showed a similar response in primary cultured VM neurons and astrocytes under oxidative stress, we performed a Western blot analysis to determine the time course of HO-1 regulation in cells exposed to 100 μmol/L of MPP^+^ or 5 μg/mL of α-synuclein. As shown in Fig. 1, HO-1 levels were elevated 12 h after MPP^+^ exposure, but they did not differ significantly from the control. A robust regulation (1.95-fold relative to the control) was observed 24 h after MPP^+^ exposure. However, MPP^+^ regulation of HO-1 was time-dependent in astrocytes, beginning at 1 h, increasing gradually to the maximal levels (approximately 1.75-fold relative to the control) at 12 h, and declining after 24 h. A similar tendency was observed in cells with α-synuclein exposure (Fig. 2). These results indicate that HO-1 responded earlier in astrocytes than in neurons under oxidative stress.

### A prompt HO-1 response contributed to less vulnerability of astrocytes compared with neurons

**Figure 3:**
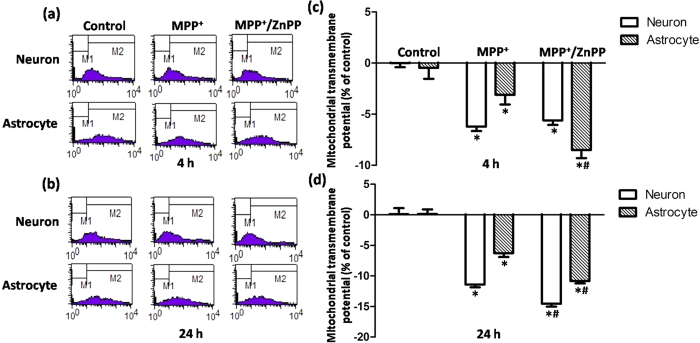
HO-1 inhibition augmented MPP^+^-induced ΔΨm collapse in both neurons and astrocytes. (**a**,**b**) Representative examples of the fluorometric assay of ΔΨm of different groups. MPP^+^ incubation induced a more severe ΔΨm reduction in neurons compared with astrocytes. A more obvious ΔΨm reduction was observed in astrocytes co-administered with ZnPP at 4 h (**a**). ΔΨm reduction was further aggravated with ZnPP co-administration for 24 h in both neurons and astrocytes (**b**). (**c**,**d**) Statistical analysis. The data are presented as the mean ± S.E.M. of more than 6 independent experiments. ^*^P < 0.05, compared with control; ^#^*P* < 0.05, compared with MPP^+^ group.

**Figure 4:**
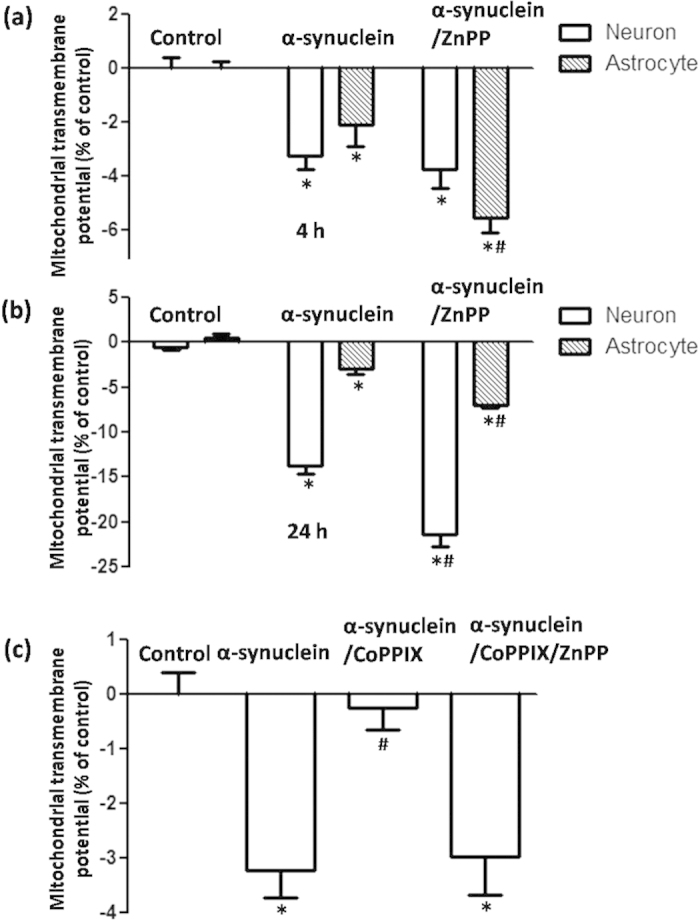
Effects of HO-1 modulation on ΔΨm collapse induced by α-synuclein exposure. (**a**) α-synuclein incubation induced a ΔΨm reduction in both neurons and astrocytes. A more obvious ΔΨm reduction was observed in astrocytes with ZnPP co-administration at 4 h. (**b**) ΔΨm reduction was further aggravated with ZnPP co-administration for 24 h in both neurons and astrocytes. (**c**) A full restoration of ΔΨm was observed in neurons with α-synuclein and CoPPIX co-administration for 4 h, which could be blocked by co-administration of the HO-1 inhibitor ZnPP. ^*^P < 0.05, compared with control; ^#^*P* < 0.05, compared with α-synuclein group.

We subsequently set out to investigate whether this early HO-1 response in astrocytes was cytoprotective. MPP^+^ incubation induced a limited but significant reduction in ΔΨm in both neurons and astrocytes at 4 h (Fig. 3a,c), which is consistent with the neurotoxicity mediated by mitochondria complex I inhibition. Consistently less damage was observed in astrocytes than in neurons. However, in astrocytes, mitochondrial damage indicated by ΔΨm collapse was aggravated with MPP^+^ and ZnPP (5 μmol/L, HO-1 activity inhibitor co-administration for 4 h), which indicated that upregulated HO-1 levels were cytoprotective. This effect was not observed in neurons, which might have been due to the unchanged HO-1 levels observed at 4 h in neurons treated with MPP^+^ (Fig. 3a,c). The cytoprotective effects of HO-1 induction were further confirmed by the finding that ZnPP co-administration induced further ΔΨm reduction in both neurons and astrocytes with MPP^+^ incubation for 24 h (Fig. 3b,d), indicating that intrinsic HO-1 induction was cytoprotective in both VM neurons and astrocytes. Similar results were observed when VM neurons and astrocytes were incubated with recombinant α-synuclein for 4 h (Fig. 4a) or 24 h (Fig. 4b).

**Figure 5:**
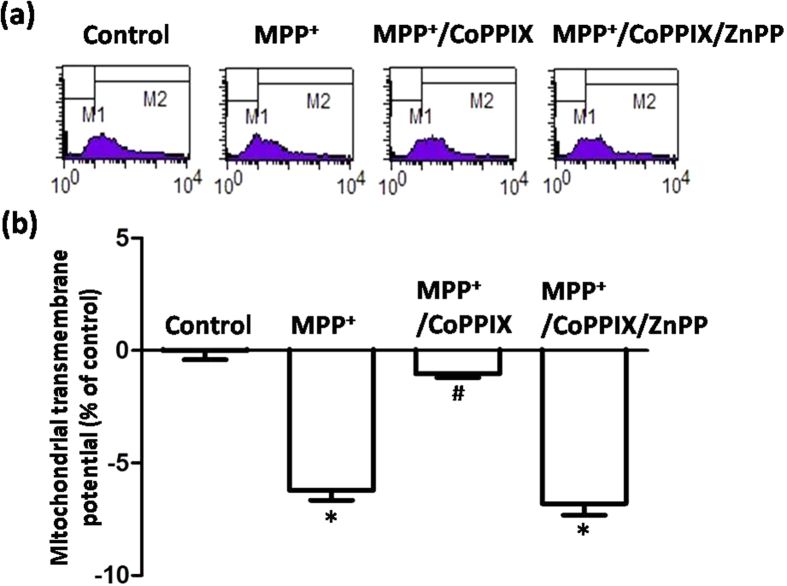
HO-1 activation rescued MPP^+^-induced ΔΨm collapse in neurons at 4 h. (**a**) Representative examples of the fluorometric assay of ΔΨm of different groups. Full restoration of ΔΨm was observed in neurons with MPP^+^ and CoPPIX co-administration for 4 h. This protective effect could be fully blocked by co-administration of the HO-1 inhibitor ZnPP (5 μg/mL). (**b**) Statistical analysis. The data are presented as the mean ± S.E.M. of more than 6 independent experiments. ^*^P < 0.05, compared with control; ^#^*P* < 0.05, compared with MPP^+^ group.

Considering that prompt induction of HO-1 could exert cytoprotective effects under oxidative stress, we applied CoPPIX (5 μmol/L), a classical HO-1 activator, to increase HO-1 levels in neurons that did not respond after a 4 h incubation with MPP^+^ or α-synuclein. As expected, co-administration of CoPPIX rescued ΔΨm collapse, which could subsequently be blocked by the HO-1 inhibitor ZnPP (Figs 4c and 5). This result indicated that sufficient expression of HO-1 plays a role in cellular antioxidation and further supports the idea that an early HO-1 response in astrocytes compared with neurons might contribute to less vulnerability of these cells.

### Overexpressed HO-1 conferred cytoprotective effects on MPP^+^-induced mitochondrial damage in VM astrocytes but not in neurons

**Figure 6:**
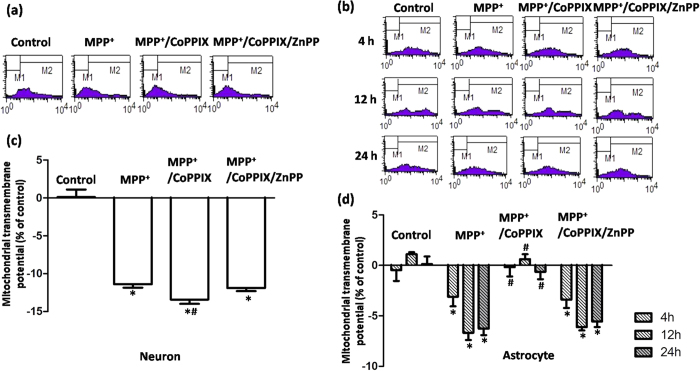
HO-1 activation exerted opposite effects on MPP^+^-induced ΔΨm collapse between neurons and astrocytes at 24 h. (**a**,**b**) Representative examples of the fluorometric assay of ΔΨm of different groups. ΔΨm in neurons was further reduced in neurons with MPP^+^ and CoPPIX co-administration for 24 h. This deleterious effect of CoPPIX could be restored to the same levels as the MPP^+^ through co-administration of the HO-1 inhibitor ZnPP (**a**). For astrocytes, a full restoration of ΔΨm was consistently observed in cells with MPP^+^ and CoPPIX co-administration for 4 h, 12 h, and 24 h, which could be blocked by co-administration with ZnPP (**b**). (**c**,**d**) Statistical analysis. The data are presented as the mean ± S.E.M. of more than 6 independent experiments. ^*^P < 0.05, compared with control; ^#^*P* < 0.05, compared with MPP^+^ group.

**Figure 7:**
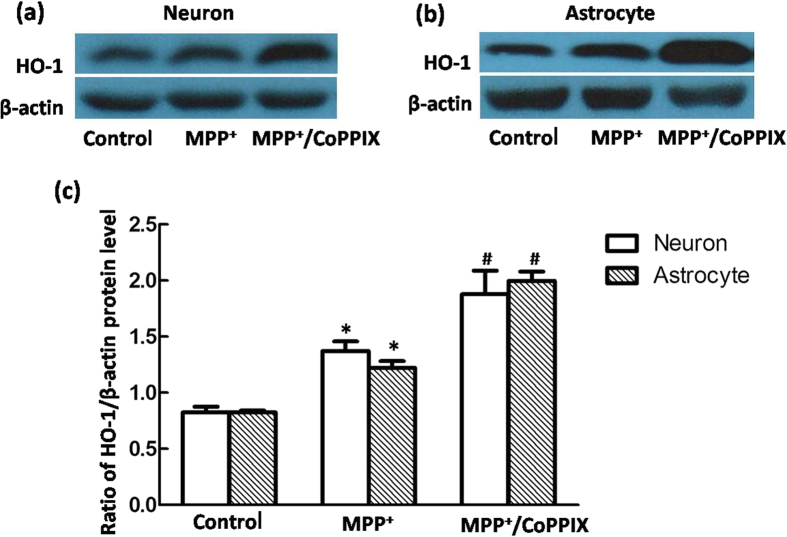
HO-1 protein levels were upregulated to the same extent with MPP^+^ and CoPPIX co-administration in VM neurons (24 h) and astrocytes (12 h). (**a**,**b**) Western blots were used to detect HO-1 protein levels in neurons (**a**) or astrocytes (**b**) with MPP^+^ and CoPPIX co-administration. A more robust HO-1 upregulation was detected in neurons at 24 h, as well as in astrocytes at 12 h, compared with the sole MPP^+^ treated group. β-Actin was used as a loading control. (**c**) Statistical analysis. The data are presented as the ratio of HO-1 to β-actin. Each bar represents the mean ± S.E.M. of more than 6 independent experiments. ^*^P < 0.05, compared with control. ^#^*P* < 0.05, compared with MPP^+^.

HO-1 did not always exert cytoprotective effects in neurons, and ΔΨm was further reduced rather than restored in neurons that underwent co-incubation with CoPPIX and MPP^+^ for 24 h (Fig. 6a,c), indicating that over-activation of HO-1 aggravated neuronal oxidative damage induced by toxins. Conversely, CoPPIX-induced HO-1 activation consistently exerted cytoprotective effects in astrocytes. Full restoration of mitochondrial functions was observed in astrocytes co-incubated with CoPPIX and MPP^+^ for 4 h, 12 h or 24 h. This protective effect was evident even at the time point (12 h) at which the astrocyte HO-1 response peaked (Fig. 6b,d), and it was distinct from the deleterious effect observed at 24 h, when the neuronal HO-1 response peaked (Fig. 6a,c). We subsequently treated cells with CoPPIX and MPP^+^ until they reached their respective peak HO-1 responses (neurons 24 h; astrocytes 12 h). Similar HO-1 levels were detected in both neurons and astrocytes after co-incubation for 24 h or 12 h with CoPPIX and MPP^+^ (Fig. 7), indicating that the different responses of neurons and astrocytes were not due to the unequal HO-1 levels.

### MtFt buffering capacity contributes to a difference in vulnerability to HO-1 between astrocytes and neurons

**Figure 8:**
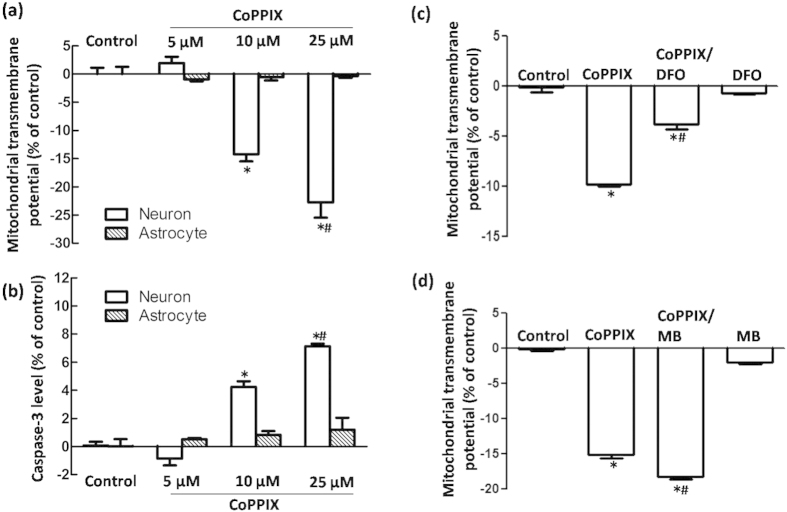
Effects of HO-1 activation on ΔΨm and caspase-3 activation in neurons and astrocytes at 24 h. (**a**,**b**) Flow cytometry was used to detect ΔΨm and caspase-3 activation in neurons and astrocytes with CoPPIX administration for 24 h. CoPPIX induced a ΔΨm reduction and caspase-3 activation in neurons in a dose-dependent manner. However, no significant changes were observed in astrocytes. The data are presented as the mean ± S.E.M. of more than 6 independent experiments. ^*^P < 0.05, compared with control. (**c**) Iron chelator DFO could partly restore the ΔΨm collapse caused by 25 μmol/L CoppIX treatment in neurons. The data are presented as the mean ± S.E.M. of more than 4 independent experiments. ^*^P < 0.05, compared with control; ^#^*P* < 0.05, compared with CoPPIX group. (**d**) MB had no effect on ΔΨm. CoPPIX (25 μmol/L) induced ΔΨm reduction. However, a significant aggravated mitochondrial damage was observed in CoppIX/MB co-treated group. The data are presented as the mean ± S.E.M. of 5 independent experiments. ^*^P < 0.05, compared with control; ^#^*P* < 0.05, compared with CoPPIX group.

We examined why HO-1 activation protected astrocytes from MPP^+^-induced oxidative stress but augmented damage resulting from oxidative injury in neurons. We first confirmed that CoPPIX induced mitochondrial damage in neurons in a dose-dependent manner, as indicated by ΔΨm collapse and caspase-3 activation. However, this damage was non-detectable in astrocytes with administration of up to 25 μmol/L CoPPIX (Fig. 8a,b). As a double-edged sword, the CO and biliverdin produced by HO-1 catabolism of heme serve as powerful reductive byproducts, but redox-active iron might be the key player in oxidative injury^24^. This was supported by the results that iron chelator DFO could partly block the deteriorated effects of CoPPIX; on the contrary, CO scavenger MB aggravated CoPPIX induced mitochondrial damage in neurons.

**Figure 9:**
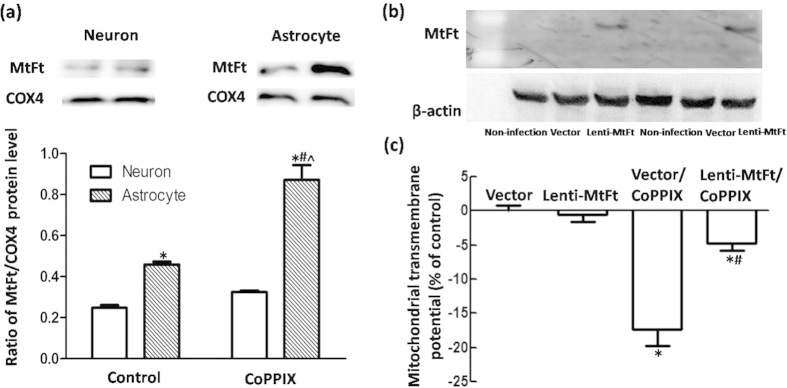
MtFt overexpression rescued ΔΨm collapse in neurons with CoPPIX administration. (**a**) Western blots were used to detect MtFt protein levels in neurons and astrocytes with CoPPIX administration for 24 h. MtFt levels were higher and more robustly upregulated in astrocytes than in neurons. The data are presented as the ratio of MtFt to COX4. Each bar represents the mean ± S.E.M. of more than 6 independent experiments. ^*^P < 0.05, compared with neurons without CoPPIX treatment; ^#^*P* < 0.05, compared with astrocytes without CoPPIX treatment; ^^^*P* < 0.05, compared with neurons with CoPPIX treatment. (**b**) Western blots were applied to detect MtFt protein levels in neurons with pLenti-Ubc-MtFt or pLenti-Ubc-MCS transduction. (**c**) Flow cytometry was applied to detect ΔΨm in different groups. Lentiviral transduction (pLenti-Ubc-MtFt or pLenti-Ubc-MCS) had no effect on ΔΨm. CoPPIX (25 μmol/L) induced ΔΨm reduction in the lentiviral vector group. However, a significant restoration was observed in the MtFt over-expression group. The data are presented as the mean ± S.E.M. of more than 4 independent experiments. ^*^P < 0.05, compared with lentivector group; ^#^*P* < 0.05, compared with lentivector/CoPPIX group.

HO-1 did not affect the level of the classical iron sequestration molecule ferritin^31^, and therefore, we investigated MtFt regulation in neurons and astrocytes with HO-1 activation. A higher basal MtFt level was detected in astrocytes than in neurons. Significant upregulation of MtFt levels was observed in both neurons and astrocytes after 5 μmol/L CoPPIX incubation, which indicated that HO-1 activity promotes iron trapping within mitochondria. However, a more robust MtFt expression (190% compared with the control) was detected in astrocytes than in neurons (131% compared with the control), providing evidence that a larger iron buffering system was present (Fig. 9a). We then evaluated whether MtFt overexpression mediated by lentivirus might be protective against CoPPIX-induced toxicity in neurons. As expected, a significant restoration of ΔΨm was observed in 25 μmol/L CoPPIX-treated neurons with MtFt overexpression compared with that of the lentivector control (Fig. 9b,c).

## Discussion

To our knowledge, this is the first report to reveal that HO-1 (which has been recognized as a dynamic sensor of cellular oxidative stress and likely arbiter of tissue redox homeostasis) produced different responses in neurons and astrocytes under the condition of neurotoxicity related to PD. We also demonstrated that HO-1 activation in neurons might aggravate oxidative damage, it but exerts protective effects in astrocytes. This difference might be due to unequal changes in MtFt between these two cell types.

Despite our limited knowledge of the etiology of PD, it is accepted that oxidative stress causes dopaminergic neuron death because the levels of oxidized lipids, DNA and proteins are increased, and glutathione levels are decreased in the SNpc of PD patients^6^,^7^,^32^,^33^. Oxidative stress might be caused by overproduction of reactive free radicals or impairment of the cellular antioxidant defense mechanisms^15^. The ability to combat oxidative stress is limited in neurons, but it is powerful in astrocytes^13^,^14^,^15^. A postmortem study demonstrated that a fraction of GFAP-positive astrocytes expressed HO-1 in the substantia nigra of PD patients (77.1 ± 12.3) at a level that was significantly greater than that observed in the substantia nigra of control subjects (18.7 ± 7.1; P = 0.0015). However, neuronal (both dopaminergic and non-dopaminergic) HO-1 immunoreactivity was moderate or faint, and few differences were observed in HO-1 expression between the two groups^27^. Thus, predominant HO-1 expression in astrocytes might explain the discrepancy between neurons and astrocytes in vulnerability to oxidative stress. In the current study, we demonstrated that in primary cultured astrocytes exposed to MPP^+^ or recombinant α-synuclein, HO-1 showed a time-dependent response that occurred earlier than in neurons, further supporting the idea that HO-1 was more efficiently induced in astrocytes than in neurons. This finding is consistent with a previous report in which Nrf2, a key transcriptional regulator of HO-1, was preferentially activated in astrocytes both *in vitro* and *in vivo*^15^.

A number of studies revealed that induction of HO-1 expression is an important pathway for attenuating cellular oxidative damage, and neuroprotective effects can be prevented by HO-1 inhibitors^34^,^35^,^36^,^37^. More recently, it was reported that the impaired HO-1 response was related to human PTEN-induced protein kinase 1 (PINK1) gene mutation and thus accelerated oxidative stress and neurodegeneration^38^. This finding led to the speculation that HO-1 targeting might be a candidate for neuroprotection and drug discovery in neurodegenerative diseases^20^,^39^,^40^. In the current study, mitochondrial function was determined by regulating HO-1 activity in VM neurons and astrocytes. HO-1 inhibition resulted in lower ΔΨm levels in both astrocytes and neurons with MPP^+^ or recombinant α-synuclein exposure, indicating that the intrinsic HO-1 upregulation under oxidative circumstances was cytoprotective. The deferred HO-1 levels in the early phase (4 h) in neurons provides a reasonable mechanism that can explain MPP^+^- or α-synuclein-induced mitochondrial damage in these cells because of HO-1 insufficiency. This observation is further supported by the observation that inducing HO-1 expression could fully resist mitochondrial impairment at this time point.

These findings suggest that HO-1 targeting could be beneficial for decreasing neuronal vulnerability to attack by oxidative stimuli. However, this situation is not always the case. Significant cytotoxicity rather than cytoprotection was observed with HO-1 induction for 24 h in VM neurons with MPP^+^ exposure. At this time point, the intrinsic HO-1 response was obviously triggered by MPP^+^ insult. In contrast, we observed that HO-1 induction in astrocytes showed consistent protective effects on mitochondrial function, even when MPP^+^ triggered the maximum HO-1 response. To exclude the possibility that the discrepancy was due to differential HO-1 induction between neurons and astrocytes, we first demonstrated that HO-1 protein levels in cells with MPP^+^ and CoPPIX co-exposure were exactly the same between the two cell types. This result indicated that overactivation of HO-1 in neurons might be deleterious. Indeed, despite the extensive neuroprotective effects afforded by HO-1, the action of this enzyme has proven detrimental in several models of CNS injury and disease^41^,^42^,^43^. Cytoprotection is believed to be conveyed by the antioxidant products biliverdin, bilirubin and CO. In contrast, the pro-oxidant product ferrous iron liberated by heme degradation might exacerbate oxidative stress. We hypothesized that the enhanced neuronal vulnerability is due to a decreased tolerance to iron generated by CoPPIX exposure and HO-1 activation.

It is well demonstrated that even if ferrous iron is elevated to the same degree in neurons and astrocytes, only neurons are injured and astrocytes are unaffected, indicating a more efficient iron buffering system in astrocytes than in neurons^44^,^45^. However, intracellular ferritin levels, and transferrin receptors and iron regulatory proteins as well, were unchanged in HO-1 overexpressing astrocytes, thus indicating that HO-1 had little or no effect on cytoplasmic iron metabolism^31^. Most cytoplasmic iron must enter the mitochondria to be incorporated into heme and Fe/S complexes for synthesis of enzymes, and it is well accepted that mitochondria are a predominant target for iron trafficking and metabolism. MtFt is an iron-storage protein localized in mitochondria that can sequester iron into the organelle and subsequently prevent oxidative damage via antioxidant properties derived from its ferroxidase activity^46^,^47^,^48^,^49^. In addition, we analyzed MtFt levels in neurons and astrocytes that overexpress HO-1. It is interesting that compared with astrocytes, the basal MtFt levels were much lower in neurons. HO-1 activation induced by CoPPIX triggered more dramatic upregulation in astrocytes. Considering the target of HO-1 activity, i.e., heme molecules located in mitochondria, these results showed that inadequate MtFt levels in neurons might convey a lower threshold than astrocytes to counter the detrimental effects of HO-1 because astrocytes can detoxify excess iron by increasing MtFt synthesis. This observation was further supported by our finding that MtFt overexpression rescued the toxicity induced by HO-1 overactivation in neurons.

In summary, this study provides novel insight into HO-1 contributions to the vulnerability of VM neurons and astrocytes. We revealed that HO-1 responded more promptly in astrocytes, and the deferred neuronal HO-1 response might explain why neurons are more vulnerable than astrocytes to oxidative stress in PD. Because HO-1 reactions represent a double-edged sword effect^23^, we further demonstrated that overexpressed HO-1 conferred cytoprotective effects against neurotoxin insults in astrocytes; however, this overexpression might be deleterious for neurons due to inadequate MtFt levels. Such findings might provide clues for design of appropriate astrocyte-targeted HO-1 interventions and MtFt modulations as novel neuroprotective strategies in PD therapeutics.

## Materials and Methods

### Materials

All procedures were performed in accordance with the International Guiding Principles for Biomedical Research Involving Animals. MPP^+^, recombinant α-synuclein, cobalt protoporphyrin IX (CoPPIX), zinc protoporphyrin (ZnPP), deferoxamine (DFO), methylene blue (MB) and primary antibodies against microtubule-associated protein 2 (MAP2) and GFAP were purchased from Sigma Chemical Co. (St. Louis, MO, USA). Primary HO-1 antibody was purchased from Stressgen Bioreagents (Ann Arbor, MI, USA). Primary mitochondria ferritin (MtFt) was purchased from Abcam (Cambridge, UK). Primary cytochrome c oxidase subunit IV (COX4) was purchased from Takara Biomedical Technology (Beijing, China). Rhodamine 123 was purchased from Invitrogen (Madrid, Spain). Caspase-3 Kit was from BD Biosciences, Dulbecco’s modified Eagle’s medium (DMEM)/F12, B27 and fetal bovine serum (FBS) were purchased from Gibco (Grand Island, NY, USA). All other chemicals and reagents were of the highest grade available and were obtained from local commercial sources.

### Primary cell cultures

#### Primary VM neuron culture

Primary cultures of VM neurons were obtained from embryonic Sprague–Dawley rat mesencephalon, as previously described by our lab^50^. All experimental procedures were carried out in accordance with the National Institutes of Health Guide for the Care and Use of Laboratory Animals, and were approved by Ethical Committee of the Medical College of Qingdao University. In brief, regions of the ventral mesencephalon were dissected from embryonic 14-day-old rat brains and mechanically dissociated with a pipette until the tissue was dispersed. After centrifugation at 1,000 rpm for 5 min, cells were suspended in DMEM/F12 containing 10% FBS, 100 U/mL of penicillin and 100 μg/mL of streptomycin, seeded at a density of 1.5 × 10^5^ cells/mL on poly-D-lysine-coated coverslips or 12-well culture plates, and subsequently placed in a humidified atmosphere of 5% CO_2_ at 37 °C. After incubation for 18 h, the culture medium was changed to serum-free DMEM/F12 supplemented with 2% B27. Cells were cultured for an additional 6 days before use. Neuron purity was obtained at approximately 95% based on immunofluorescence staining with a specific neuron marker for MAP2. Approximately 5% of the neurons exhibited TH positivity, and these were dopaminergic neurons. For experiments, VM neurons were treated with 100 μmol/L MPP^+^ or 5 μg/mL α-synuclein^51^ for 30 min, 1 h, 2 h, 4 h, 8 h, 12 h or 24 h to investigate time-dependent HO-1 induction. HO-1 activator CoPPIX (5 μmol/L) or HO-1 inhibitor ZnPP (5 μmol/L) were co-administered with MPP^+^ or α-synuclein for 4 h or 24 h to investigate the role of HO-1 in cellular oxidative damage.

#### Primary VM astrocyte culture

Astrocytes were isolated from the VM of newborn Wistar rats as previously described by our lab^52^. In brief, the tissues were dissected from Wistar pups aged 1–2 days, and after the meninges were removed, the tissues were transferred to a tube containing cold D-Hank’s solution. The pure tissue was mechanically dissociated and digested by incubation with 0.1% trypsin for 10 min at 37 °C. The reaction was stopped by the addition of complete medium (DMEM/F12, 10% FBS, 100 U/mL of penicillin and 100 μg/mL of streptomycin). The tissues were mechanically digested again by pipetting until full dissociation into single cells was accomplished. After centrifugation at 1,000 rpm for 5 min, the pellets were resuspended in complete medium and plated in poly-D-lysine-coated 150-cm^2^ flasks (5 brains/flask). After approximately 8–10 days, the confluent culture was placed horizontally on a shaker platform with medium covering the cells, shaken at 250 rpm for 18 h, and the medium that contained microglia was removed. The attached cells were trypsinized and re-plated to 6-well plates at a density of 3 × 10^5^ cells/mL. Cells were subsequently cultured until they reached 80% confluence for further experiments as described above for VM neurons. These pharmacological treatments did not affect the viability of neurons or astrocytes (data not shown). Astrocyte purity was greater than 95% based on immunofluorescence staining with a specific astrocyte marker GFAP.

### Western blots

VM neurons or astrocytes were treated as described above, washed with cold PBS, and lysed with lysis buffer containing 50 mmol/L Tris HCl, 150 mmol/L NaCl, 1% Nonidet P-40, 0.5% sodium deoxycholate, 1 mmol/L EDTA, 1 mmol/L phenylmethylsulfonyl fluoride (PMSF), and protease inhibitors (pepstatin 1 μg/mL, aprotinin 1 μg/mL, leupeptin 1 μg/mL) for 30 min on ice, and the insoluble material was removed by centrifugation (12,000 rpm, 20 min, 4 °C). Protein concentration was determined by the Bradford assay kit (Bio-Rad Laboratories, Hercules, CA). A total of thirty micrograms of protein was separated using 10% SDS-polyacrylamide gels and transferred to NC membranes. After blocking with 5% non-fat milk at room temperature for 1 h, the membranes were incubated with rabbit anti-rat primary antibodies against HO-1 (1:2000) or MtFt (1:1000) for 2 h at 4 °C. Anti-rabbit secondary antibody conjugated to horseradish peroxidase was used at 1:10,000 (Santa Cruz Biotechnology, Santa Cruz, CA). β-actin and COX4 were detected using mouse anti-β-actin monoclonal antibody (1:8000) according to a similar procedure that ensured equal samples of total protein and mitochondrial protein, respectively. Cross-reactivity was visualized using ECL Western blot detection reagents and analyzed via scanning densitometry using a UVP BioDoc-It Imaging System (UVP, Upland, USA).

### Detection of mitochondria transmembrane potential (ΔΨm) and caspase-3 activation

MPP^+^- or α-synuclein-induced oxidative damage was indicated by changes in ΔΨm for VM neurons or astrocytes using flow cytometry (Becton Dickinson, USA) as previously described^50^,^53^. Cells were co-treated with MPP^+^ or α-synuclein and CoPPIX or ZnPP for 4 h or 24 h and incubated in HBS with rhodamine 123 in the dark at a final concentration of 5 μmol/L for 20 min at 37 °C. After washing three times with HEPES-buffered saline (HBS: 10 mmol/L HEPES, 150 mmol/L NaCl, pH 7.4), cells were re-suspended in 1 mL HBS. For analysis, excitation and emission wavelengths of 488 and 525 nm, respectively, were used to assess 10,000 cells from each sample. The results are presented as FL1-H (Fluorescence 1-Histogram), setting the gated regions M1 and M2 as markers to observe the changing levels of fluorescence intensity using Cellquest Software. For dose-dependent CoPPIX effects on mitochondrial damage, neurons or astrocytes were treated with different concentrition CoppIX (5, 10, 25 μmol/L) for 24 h and then collected for detection of ΔΨm and caspase-3 activation. DFO (100 μmol/L) or MB (0.01 μmol/L) were co-administrated with 25 μmol/L CoPPIX to evaluate the effects of HO-1 catalyzing byproducts. Caspase-3 activation was measured following Caspase-3 Kit staining protocol. The results are presented as FL2-H (Fluorescence 2-Histogram), setting the gated regions M1 and M2 as markers to observe the changing levels of fluorescence intensity using Cellquest Software.

### Lentiviral transduction in primary VM neurons

pLenti-Ubc-MtFt and pLenti-Ubc-MCS were reconstructed with rat MtFt cDNA by Obio (Obio Technology, Shanghai, China). To determine the transduction efficiency in primary neurons, GFP expression was examined by fluorescence microscopy (ZEISS, German) at different multiplicities of infection (MOIs) on day 3 after infection. Western blotting was used to evaluate MtFt levels. Primary VM neurons were treated with CoPPIX (25 μmol/L) for 24 h on day 3 after infection by pLenti-Ubc-MtFt or pLenti-Ubc-MCS and subsequently collected for ΔΨm detection.

### Statistical analysis

The data are presented as the mean ± S.E.M. Differences between mean values in two groups were compared using unpaired t-tests. One-way analysis of variance followed by the Student-Newman-Keuls test was used to compare mean values among three or more groups. A probability value of *P* < 0.05 was considered statistically significant.

## Additional Information

**How to cite this article**: Yu, X. *et al.* Differences in vulnerability of neurons and astrocytes to heme oxygenase-1 modulation: Implications for mitochondrial ferritin. *Sci. Rep.* **6**, 24200; doi: 10.1038/srep24200 (2016).
